# Population- and Variant-Based Genome Analyses of Viruses from Vaccine-Derived Rabies Cases Demonstrate Product Specific Clusters and Unique Patterns

**DOI:** 10.3390/v12010115

**Published:** 2020-01-17

**Authors:** Sten Calvelage, Marcin Smreczak, Anna Orłowska, Conrad Martin Freuling, Thomas Müller, Christine Fehlner-Gardiner, Susan Nadin-Davis, Dirk Höper, Paweł Trębas

**Affiliations:** 1 Institute of Diagnostic Virology, Friedrich-Loeffler-Institut, 17493 Greifswald-Insel Riems, Germany; sten.calvelage@fli.de (S.C.); dirk.hoeper@fli.de (D.H.); 2 Department of Virology, National Veterinary Research Institute, 24-100 Puławy, Poland; marcin.smreczak@piwet.pulawy.pl (M.S.); anna.orlowska@piwet.pulawy.pl (A.O.); pawel.trebas@piwet.pulawy.pl (P.T.); 3 Institute of Molecular Virology and Cell Biology, Friedrich-Loeffler-Institut, WHO Collaborating Centre for Rabies Surveillance and Research, OIE Reference Laboratory for Rabies, 17493 Greifswald-Insel Riems, Germany; thomas.mueller@fli.de; 4 National Reference Centre for Rabies, Ottawa Laboratory–Fallowfield, Canadian Food Inspection Agency, Ottawa, ON K2H 8P91, Canada; christine.fehlner-gardiner@canada.ca (C.F.-G.); nadindavis@gmail.com (S.N.-D.)

**Keywords:** rabies, vaccine, next generation sequencing, population analysis

## Abstract

Rabies in wildlife has been successfully controlled in parts of Europe and North America using oral rabies vaccination, i.e., the distribution of baits containing live-attenuated virus strains. Occasionally, these vaccines caused vaccine virus-induced rabies cases. To elucidate the mechanisms of genetic selection and the effect of viral populations on these rabies cases, a next generation sequencing approach as well as comprehensive data analyses of the genetic diversity of Street Alabama Dufferin (SAD) and ERA vaccine virus strains and vaccine-induced rabies cases from Canada and several European countries were conducted. As a result, twelve newly generated sets of sequencing data from Canada and Poland were added to a pool of previously investigated samples. While the population-based analysis showed a segregation of viruses of ERA vaccine-induced rabies cases from those of SAD Bern original (SAD Bern_orig_)-derived rabies cases, the in-depth variant analysis revealed three distinct combinations of selected variants for the ERA vaccine-induced cases, suggesting the presence of multiple replication-competent haplotypes in the investigated ERA-BHK21 vaccine. Our findings demonstrate the potential of a deep sequencing approach in combination with comprehensive analyses on the consensus, population, and variant level.

## 1. Introduction

Vaccination programs are one of the most effective means of controlling infectious diseases [1] and with the development of oral vaccines and bait delivery systems, the elimination of diseases circulating in wildlife populations has become a realistic possibility. The large-scale oral rabies vaccination (ORV) campaigns that have eliminated fox-mediated rabies from Western Europe and North America [2,3] and substantially reduced disease incidence in central Europe [2] are pre-eminent examples for the success of such control programs.

ORV programs in foxes are aimed at increasing herd immunity in the target population using oral rabies vaccines distributed into the environment. Over the past four decades several oral rabies vaccines, mainly live replication-competent attenuated rabies virus vaccines, have been successfully used in ORV campaigns [4]. In Canada for example, the ERA-BHK21vaccine virus strain, a derivative of the cell culture adapted vaccine virus strain Street Alabama Dufferin (SAD) [5,6], was the only live attenuated vaccine deployed in fox ORV campaigns [7]. In Europe, with the exception of a recombinant vaccinia virus expressing the rabies virus glycoprotein, all constructs have been based on live attenuated rabies virus strains [4], derived from the SAD Bern original (SAD Bern_orig_) vaccine virus strain, a successor of the ERA strain [2,8]. While all these vaccines have been highly efficient in fox rabies control, the first generation of SAD-derived vaccines demonstrated residual pathogenicity in non-target species particularly in rodents [9,10,11,12]. Although several cases of vaccine virus-induced rabies were observed even in species other than rodents over the course of vaccination campaigns in a number of countries, including Germany [1], Austria [1], Slovenia, Romania [13], Poland, and Canada [7], such cases were without epidemiological relevance [1,7,8].

While previous analyses using high-throughput sequencing approaches revealed substantial genetic heterogeneity within commercial SAD-derived oral rabies virus vaccines [14], the sub-consensus genetic heterogeneity of viruses isolated from these vaccine virus-induced rabies cases on the contrary revealed nearly clonal genotypes, indicating the presence of a strong bottleneck during infection [15].

In this study, we attempted to further analyze and elucidate the mechanisms of genetic selection using a combined deep sequencing, variant, and haplotype analysis of a large set of vaccine-induced rabies cases that included additional SAD- and ERA-induced rabies cases from Poland and Canada alongside vaccine virus batches. With this comprehensive approach, we could demonstrate the utility of this approach for identity and genetic stability (revision to virulence) testing of vaccines with a heterogeneous genetic background. Additionally, we were interested to know whether viruses from vaccine-induced rabies cases differ from virulent field rabies viruses (RABV) at a population level.

## 2. Materials and Methods

### 2.1. Samples

In this study, original brain material of additional ERA-BHK21 and SAD Bern_orig_-derived vaccine-induced rabies cases from Canada [7] and Poland, respectively, were analyzed (Table 1). In addition, new batches of the attenuated rabies virus vaccine SAD Bern (Lysvulpen) were obtained and included in the analysis (Table 2). In order to check for validity of the PCR based and Illumina generated sequence reads for the use of the methods to be applied, we included datasets of two case samples (C/CAN/1994/MB and C/CAN/1996/B) as well as a dataset of the ERA/lot16 vaccine batch already sequenced on an IonTorrent PGM platform from a previous study [15].

### 2.2. Nucleic Acid Extraction, Sample Processing, and Sequencing

Samples were processed according to two different methodical approaches. Methodology 1 corresponds to the published protocol of Wylezich and colleagues [16]. Briefly, for sample disintegration prior to nucleic acid extraction, 20 mg tissue material or 200 µL of the vaccine virus preparation from the blister were cryofractured using the cryoPREP CP02 (Covaris, Brighton, United Kingdom). The pulverized material was lysed with pre-heated (56 °C) buffer AL (Qiagen, Hilden, Germany). A threefold volume of Trizol was added and RNA extracted using the RNeasy Mini Kit (Qiagen) followed by on-column DNase I digestion (Qiagen). Subsequently, the obtained RNA was converted into double stranded cDNA using a combination of SuperScript™ IV First-Strand cDNA Synthesis System (Invitrogen/Thermo Fisher Scientific, Waltham, MA, USA) and NEBNext^®^ Ultra™ II Non-Directional RNA Second Strand Synthesis Module (New England Biolabs, Ipswich, MA, USA). The generated cDNA was fragmented using the Covaris M220 (Covaris) and subsequently converted to Ion Torrent compatible libraries using the GeneRead L Core Kit (Qiagen) and IonXpress Barcode Adaptors (Thermo Fisher Scientific, Waltham, MA, USA) followed by size selection as described [16]. After quality control (Agilent 2100 Bioanalyzer; High Sensitivity DNA Kit, Agilent Technologies, Santa Clara, CA, USA) and quantification (KAPA Library Quantification Kit—Ion Torrent PGM Uni; KAPA Biosystems/Roche, Basel, Switzerland), the libraries including the Ion S5 Calibration Standard were sequenced on an Ion 530 chip with an Ion S5 XL System (Thermo Fisher Scientific) in 400 bp-mode according to the manufacturer’s instructions.

Methodology 2 followed sample preparation as described before [17]. In short, RNA was prepared using a protocol combining Trizol and a semi-automated MagMax™ (Applied Biosystems/Thermo Fisher Scientific, Waltham, MA, USA) extraction system. Subsequently, dsDNA for input into library preparation was generated by reverse-transcription polymerase chain reaction (RT-PCR), yielding overlapping amplicons of the RABV genome (Tables S1 and S2, Supplementary File). The generated amplicons were individually quantified for each sample utilizing a Qubit fluorometer (ThermoFisher Scientific) and pooled equimolarly followed by library generation using the Nextera XT kit (Illumina, San Diego, CA, USA). Sequencing was performed with an Illumina MiSeq instrument generating 250 base paired end reads.

### 2.3. Population-Based Analysis, Sequence Assembly, and In-Depth Variant Analysis

Data analysis was performed essentially as described before with the respective published data included [14,15] (Tables S3–S6, Supplementary File). After trimming for quality of reads (all datasets), the dataset sizes were adjusted to match the size of the published datasets [14,15]. All sequence reads were mapped along a strict majority consensus sequence and its reverse complement (454 Sequencing System Software v3.0; Roche) that was previously generated and introduced via a MAFFT (multiple sequence alignment using fast Fourier transform) alignment of published SAD-related sequences [15]. Using R (version 3.6.0) and RStudio (version 1.2.1335) in combination with additional packages (Table 3), base frequencies of the forward and reverse mapping were calculated for each position of the reference sequence. Subsequently, the data were corrected for strand-bias and pairwise Manhattan distances were calculated for each population. After non-linear multidimensional scaling, the distances were displayed in 2-dimensional plots [15]. For reasons of simplification, previously sequenced in vitro selected attenuated rabies vaccine strains, i.e., P5/88, VA1, SAG2, SAD B19_CS_, and SAD B19_P1_ (Table S3, Supplementary File), were excluded to achieve a more convenient presentation of the SAD Bern_orig_-derived vaccine-induced rabies cases analyzed in this study.

Full genome sequences of all samples listed in Table 1 and Table 2 were obtained by de-novo assembly of full or partial matching reads from the forerun mapping (454 Sequencing System Software v3.0; Roche) and submitted to the European Nucleotide Archive (ENA) under the study accession PRJEB35810.

For in-depth variant analysis, strand-bias corrected data of base frequencies were used as the basis for comparison of single nucleotide variants between vaccine virus strains and their induced rabies cases. Here, all nucleotide variants with a variant frequency of at least five percent (Table S7, Supplementary File) were considered. For sample datasets generated with both IonTorrent and Illumina (C/CAN/1994/MB—C/CAN/1994N6762M, C/CAN/1996/B—C/CAN/1996N5367, and ERA/lot16), variants were taken into account if they were present in both datasets and their mean variant frequency was equal or above five percent. In parallel, fully or partially mapped sequence reads were analyzed by Geneious Prime (2019.2.3; build 2019-09-24) for an additional variation analysis using standard settings and a minimum variant frequency threshold of 0.05 to confirm positions and frequencies of single nucleotide variants found in the strand-bias corrected dataset. Noteworthy, the populations determined for samples C/CAN/1994/MB—C/CAN/1994N6762M and C/CAN/1996/B—C/CAN/1996N5367 from Ion Torrent and Illumina data were nearly identical, putting the related viruses in close proximity in the distance analysis.

## 3. Results

To expand the knowledge and understanding of the dynamics of ERA-BHK21 and SAD Bern_orig_-derived oral rabies vaccines, twelve additional datasets of vaccine-induced rabies cases and their corresponding vaccines were investigated (Table 1 and Table 2).

### 3.1. Viruses from Vaccines Form Product Specific Clusters in Population Analysis

**Figure 1:**
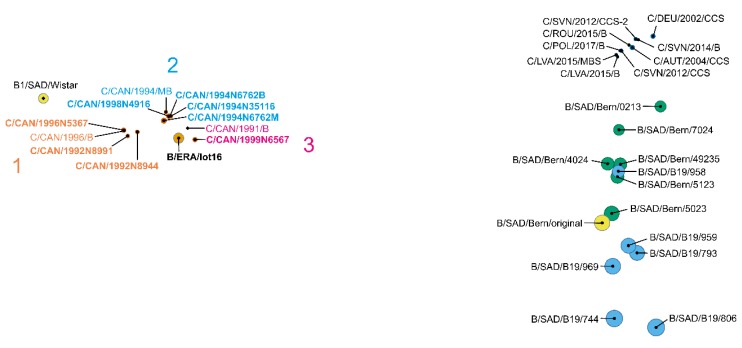
Population-based analysis of ERA-BHK21 (**left**) and SAD Bern_orig_-derived vaccine batches (B/…) (**right**) and viruses of their vaccine-induced rabies cases (C/…) displayed by a fitted pairwise Manhattan distances plot. Data sets of samples that were added in this study (Table 1 and Table 2) are highlighted in bold, whereas data sets of vaccine batches and their related induced rabies cases are displayed in different colors: blue—SAD B19 vaccine batches; green—SAD Bern vaccine batches, dark blue—SAD Bern_orig_-derived vaccine-induced rabies cases; orange—ERA-BHK21 vaccine batch; and dark orange—ERA-derived vaccine-induced rabies cases (orange/dark orange). For the ERA vaccine-induced rabies cases, three distinct combinations of single nucleotide variants were selected that originate from the progenitor ERA-BHK21 vaccine (see Section 3.2), forming groups of samples with specific differences that are represented by the colors of the respective sample names (1–3).

Overall, the distance plot generated from the population data segregates the different oral rabies vaccine viruses into two major clusters, i.e., ERA-BHK21- and SAD Bern_orig_-derived vaccines (Figure 1), confirming previous observations [14,15]. When additional SAD Bern (Lysvulpen) batches were included, the SAD Bern_orig_-derived cluster appeared to form two sub-clusters. While the majority of SAD B19 and SAD Bern vaccine batches clearly separated into these sub-clusters, one batch of each group was dislocated. Namely, batch B/SAD/B19/958 clustered with SAD Bern (Lysvulpen) vaccine batches, whereas vaccine batch B/SAD/Bern/0213 was shifted to the vaccine-induced rabies cases (Figure 1) as previously described [15].

Population analysis of the viruses isolated from vaccine-induced rabies cases revealed two distinct patterns. Those viruses that were associated with SAD Bern_orig_-derived oral rabies vaccines (including the newly analyzed Polish case C/POL/2017/B) did not cluster with the related vaccines as shown before [15]. In contrast, the viruses from the ERA vaccine-induced rabies cases all clustered closely together with the ERA-BHK21 vaccine batch (B/ERA/lot16, Figure 1). To further elucidate the observed differences, we conducted an in-depth analysis of the relations between viruses from SAD Bern_orig_- and ERA vaccine-induced rabies cases to their progenitor vaccines.

### 3.2. ERA-Derived Subclusters Comprise Unique Patterns of Base Exchanges

**Figure 2:**
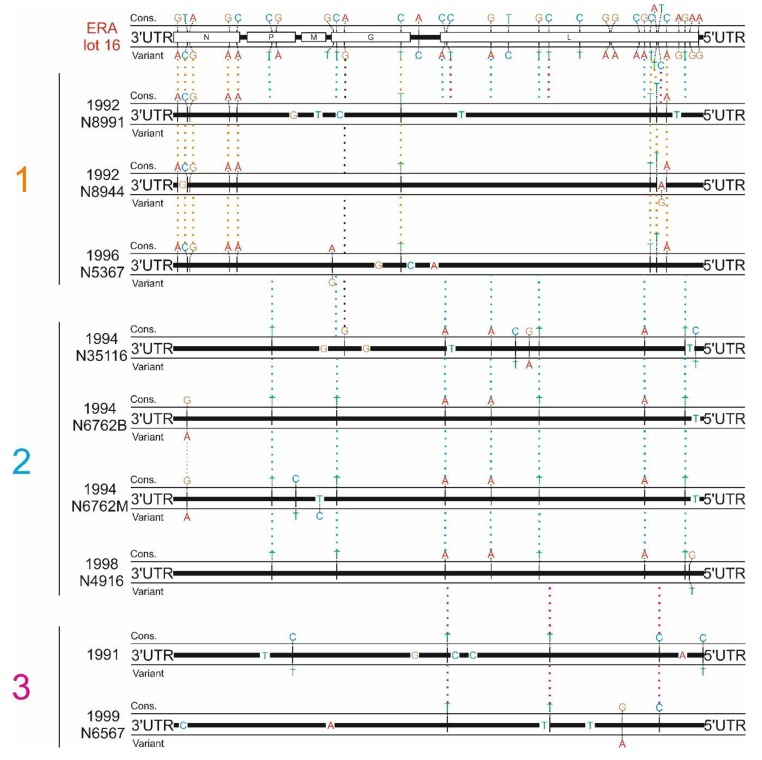
Schematic illustration of all variants found for the ERA vaccine-induced rabies cases as well as nucleotide exchanges between viruses from vaccine-induced rabies cases and the ERA-BHK21 vaccine virus strain. Colored dotted lines display differences at the consensus level for viruses from vaccine-induced rabies cases that derived from selected combinations of single nucleotide variants (SNVs) found in the progenitor ERA-BHK21 vaccine virus (group 1–3; Table 4, for variant frequencies, see Table S7, Supplementary File). Nucleotide bases illustrated on the genome sequence represent spontaneous mutations found for viruses of vaccine-induced rabies cases. Each of these three groups were characterized by a unique set of differences at the consensus level (Table 4). The only exception was the sample C/CAN/1994N35116 which lacked one difference (position 3587, Table S7) and had one additional difference in close proximity that cannot be found in any of the other samples (position 3734, Table S7).

The ERA-BHK21 vaccine virus population displayed a high genetic heterogeneity represented by diverse single nucleotide variants distributed across the N, P, G, and L genes (Figure 2), supporting previous findings [15]. Interestingly, the in-depth variant analysis revealed distinct dependencies of the ERA vaccine-induced cases from vaccine variants, with three specific combinations of single nucleotide variants (SNVs) being selected from the progenitor population at the consensus level (Figure 2). Most of these differences were derived from single nucleotide variants (SNVs) already present in the progenitor ERA-BHK21 vaccine virus strain, while a few additional mutations at the consensus level were of spontaneous origin. While all but one of the latter mutations appeared to be unique, the selected SNVs could be found across several viruses from ERA vaccine-induced rabies cases. Thus, the analyses of viruses from the ERA vaccine-induced cases confirmed distinct combinations of SNVs. According to these combinations, three groups of viruses from ERA vaccine-induced rabies cases could be defined (Table 4, Figure 2).

Each of these groups was characterized by a unique set of differences at the consensus level ranging between three and nine genome positions that were not present in any of the other groups (Table 4, Figure 2). This suggests the presence of at least three replication-competent virus haplotypes in the progenitor ERA-BHK21 vaccine virus strain that were selected in vivo by a genetic bottleneck in affected animals [15]. However, although the limited number of ERA vaccine-induced rabies cases hampers a statistical analysis, there seems to be no spatio-temporal nor species-specific correlation among the three groups of viruses from ERA vaccine-induced rabies cases.

With 8–14 differences at the consensus level, sequence diversity of the ERA vaccine-induced rabies cases and their progenitor vaccine virus strain was relatively high compared to the viruses of the SAD Bern_orig_ rabies cases. This observation seemingly contradicts results from the population-based analysis where the ERA vaccine-induced cases clustered closely together with the ERA-BHK21 vaccine batch. However, differences at the consensus level of the viruses of ERA vaccine-induced rabies cases were already included in the progenitor vaccine population (ERA-BHK21) and likewise, in some variants, the former consensus sequence was present as a minor variant. For instance, in each of two ERA vaccine-induced rabies cases (C/CAN/1992N8944 and C/CAN/1994N6762M) one additional single nucleotide variant (A10840[G,A] and C3085[T,C]) appeared in the viral populations; in both cases, the progenitor consensus was maintained as a minor variant (Table 5).

### 3.3. SAD Bern_orig_-Derived Cases are Defined by High Sequence Identities Relative to Their Related Vaccines

Strikingly, in contrast to the ERA vaccine-induced rabies cases, for the SAD Bern_orig_-induced rabies cases no SNVs already present in the progenitor vaccines were selected, even though a higher number of variants were detected in the SAD Bern_orig_-derived vaccine virus strain (N_mean_ ≈ 47, see Table S8, Supplementary File) than in the ERA-BHK21 strain (N = 31, see Figure 2 and Table S7). Also, the consensus sequences of viruses from the SAD Bern_orig_ vaccine-induced rabies cases showed a high sequence identity to their progenitor vaccine virus strain at the consensus level, ranging from zero to four nucleotide exchanges, confirming previous studies [13,15,18].

### 3.4. Known Antigenic Sites Are Rarely Affected by Amino Acid Exchanges in Vaccine-Induced Cases

To elucidate whether the induction of disease in these vaccine-induced rabies cases was a result of mutations at known antigenic sites [19,20,21,22,23,24,25,26,27,28,29,30,31,32], all sequences were screened for nucleotide exchanges at the respective genome positions. The analysis revealed no nucleotide exchanges between the SAD Bern_orig_-derived vaccine virus strains and their induced rabies cases in any of the known antigenic sites, including SNVs on these particular positions. Furthermore, none of the antigenic sites were affected by mutations in the ERA vaccine-induced rabies cases, except for one amino acid (AA) exchange Thr178Ile at an interferon-antagonist motif in the P-gene [28] found in all members of group 2 (Table 4). Whether this single AA exchange has any influence on pathogenicity remains speculative, as the entire motif contains eleven AAs and only the deletion of segments or the complete motif was studied [28]. On the other hand, if pathogenicity is increased by this presumed haplotype of group 2, more vaccine-induced rabies cases of this variant would be expected.

### 3.5. Viruses from Vaccine-Induced Cases Can Clearly Be Distinguished from Field RABV Viruses

**Figure 3:**
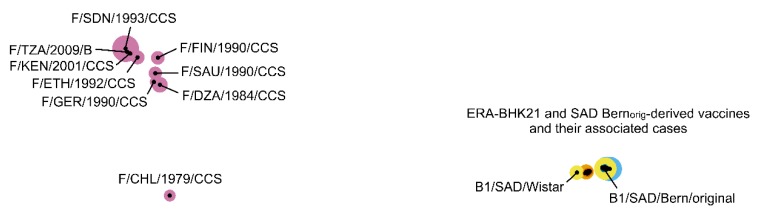
Population-based analysis of ERA-BHK21- and SAD-Bern_orig_-derived vaccine strains and viruses of their vaccine-induced rabies cases in relation to selected field RABV strains (F/…).

When selected field RABV viruses were included in the Manhattan distance matrix analysis, the attenuated vaccine virus strains and viruses from their vaccine-induced rabies cases were clearly separated from them by high distances (Figure 3). Furthermore, dimensions of the plot increased substantially compared to Figure 1, resulting in the condensation of minor differences and the formation of a single tight cluster.

## 4. Discussion

Although various studies have focused on oral rabies vaccine-induced cases [1,7,13,18,33], only a few studies have comprehensively investigated those cases and their progenitor vaccine viruses at a population level [14,15]. The latter showed a high genetic heterogeneity in different vaccine virus strains as opposed to viruses from vaccine-induced rabies cases, indicating a substantial loss in viral population diversity. In this study, we could identify two different vaccine virus-dependent patterns in the genetic markup of viruses from vaccine-induced rabies cases. This was only possible by including further samples of ERA [7] and SAD Bern vaccine-induced rabies cases in the in-depth variant analysis (Table 1). Hence, in total 20 datasets from viruses of 15 cases of vaccine-induced rabies from Europe and Canada as well as 15 related batches of the ERA-BHK21, SAD Bern, and SAD B19 vaccine strains that were collected between 1991 and 2017 [7,14,15] were considered in this analysis.

While viruses from ERA vaccine-derived cases seem to descend from different replication-competent virus haplotypes that were already present within the progenitor ERA-BHK21 vaccine virus strain (Figure 2), the absence of SNV combinations in viruses from SAD Bern_orig_ vaccine-induced rabies cases strongly suggests that only one replication-competent haplotype was present in those cases, despite a higher number of variants. One reason could be the passage history. Both ERA-BHK21 and SAD Bern_orig_ vaccine virus strains descend from the same derivative of the cell culture-adapted field virus strain, namely SAD [5,6], but were independently further developed by multiple in vitro passaging on various cell types (see [14] for a more comprehensive overview on this topic). Genetic as well as phylogenetic analyses, however, suggest that the ERA-BHK21 vaccine virus remained closer to its progenitor SAD strain [14,15]. On the other hand, the SAD Bern_orig_ vaccine virus strain was further developed into the commercial vaccine virus strains SAD Bern and SAD B19. Eventually, increased cell culture adaptation of SAD Bern and SAD B19 vaccine virus strains may have selected for only a single replication-competent haplotype. In parallel, this adaptation towards efficient replication in cell culture is favoring the development of defective interfering particles [34] that provide the background for the high genetic diversity as found by the variant analyses (Figure 1). Unfortunately, our analysis was restricted to viruses for which sufficient sequencing data was available, and thus, could not cover the SAD P5/88 vaccine-induced rabies cases [15].

As attenuated live vaccines consist of replication-competent virus particles, a major concern in their application is the potential reversion to virulence as demonstrated by vaccines for the severe acute respiratory syndrome coronavirus (SARS-CoV) [35], peste des petits ruminants virus (PPRV) [36], the avian metapneumovirus (aMPV) [37] and Rift Valley fever virus [38]. For the attenuated oral rabies vaccines investigated here, our analyses on alterations in antigenic sites showed no evidence of a reversion to virulence of viruses from vaccine-induced rabies cases either as result of the production process nor of the in vivo replication in affected animals. This is also supported by the population-based analysis conducted, which clearly separated field strains from vaccines and their induced cases (Figure 3). Rather, other factors including the individual immune status or genetic predisposition of the infected animals most likely have contributed to the development of disease. This is supported by the observation, that none of these sporadic cases resulted in onward transmission or was of epidemiological relevance [1,8].

The results of this study clearly demonstrate the benefits of utilizing deep sequencing followed by a comprehensive stepwise data analysis focusing on consensus, population, and variant level. While each of the analyses may lead to biased conclusions, only the combination allows for a holistic assessment, and thus should be standard in similar scenarios.

## Figures and Tables

**Table 1:** Overview of additional vaccine-induced rabies cases from Poland and the Ontario province of Canada analyzed in this study.

Sample ID	Animal	Origin	Date of Finding	Distributed Vaccine	Reference	Sample Material	Sequencing Method	RABV Reads	Mean Depth
C/POL/2017	Red fox	Małopolska, Poland	Dec 2017	SAD Bern	This Study	Brain	1	8286	170
C/CAN/1992N8944	Red fox	Carleton, Canada	1992	ERA	[7]	Brain	2	49,610	1138
C/CAN/1992N8991	Raccoon	Tyendinaga, Canada	1992	ERA	[7]	Brain	2	50,587	1092
C/CAN/1994N6762-B	Cow	Gloucester, Canada	1994	ERA	[7]	Brain	2	51,225	1129
C/CAN/1994N6762-M	Mouse	Gloucester, Canada	1994	ERA	[7]	Brain	2	50,403	1165
C/CAN/1994N35116	Striped skunk	Hullet, Canada	1994	ERA	[7]	Brain	2	49,319	1127
C/CAN/1996N5367	Red fox	Ops, Canada	1996	ERA	[7]	Brain	2	50,188	1090
C/CAN/1998N4916	Raccoon	Normanby, Canada	1998	ERA	[7]	Brain	2	51,239	1092
C/CAN/1999N6567	Red fox	Mulmur, Canada	1999	ERA	[7]	Brain	2	51,411	995

**Table 2:** Overview of ERA and Street Alabama Dufferin (SAD) Bern vaccine batches analyzed in this study.

Sample ID	Vaccine Strain	Batch ID	Description	Sequencing Method	RABV Reads	Mean Depth
B/ERA/lot16	ERA	lot 016	ERA-BHK21 vaccine batch from 2005	2	50,788	11,301
B/SAD/Bern/4024	SAD Bern	17/61Lj (4024)	Lysvulpen vaccine batches that were distributed in the area of Małopolska in autumn 2016, spring 2017 and autumn 2017	1	51,098	1317
B/SAD/Bern/7024	SAD Bern	17/76Lj (7024)		1	52,001	1333

**Table 3:** List of additional R packages used for the calculation and graphical representation of the Manhattan distances.

R Package	Version	Description
ape	5.3	Analyses of phylogenetics and evolution
cluster	2.0.8	Extended cluster analysis
fpc	2.2-3	Flexible procedures for clustering
ggplot2	3.2.0	Data visualizations
ggrepel	0.8.1	Non-overlapping text labels for ggplot2
grid	3.6.0	Grid graphics package
phyloseq	1.28.0	Handling and analysis of high-throughput microbiome census data
vegan	2.5-5	Community ecology package

**Table 4:** Groups of viruses from ERA vaccine-induced rabies cases showing similar patterns of nucleotide exchanges compared to the progenitor ERA-BHK21 vaccine virus strain at the consensus level.

	Samples	Host Species	Number of Group-Specific Differences
Group 1	C/CAN/1992N8944	Red fox	9
	C/CAN/1992N8991	Raccoon	
	C/CAN/1996N5367	Red fox	
	C/CAN/1996		
Group 2	C/CAN/1994N35116	Striped skunk	7
	C/CAN/1994N6762B	Cow	
	C/CAN/1994N6762M	Cow (M-passage) ^1^	
	C/CAN/1994		
	C/CAN/1998N4916	Raccoon	
Group 3	C/CAN/1991	Striped skunk	3
	C/CAN/1999N6567	Red fox	

^1^ Mouse-passaged virus originating from C/CAN/1994N6762-B.

**Table 5:** Viruses of ERA vaccine-induced rabies cases that maintained the vaccine consensus in the form of an SNV.

Sample ID	Position *	Consensus Vaccine-Induced Case	Variant Vaccine-Induced Case (Consensus Vaccine)	Frequency of SNVs in the Vaccine-Induced Case
CAN/1992N8944	10,840	A	G	33.7%
CAN/1994N6762-M	3085	T	C	31.9%

* Nucleotide position relative to the sequence of the ERA-BHK21 vaccine batch B/ERA/lot16 full genome.

## Supplementary Materials

Supplementary materials can be found at https://www.mdpi.com/1999-4915/12/1/115/s1.
