# Supplementary material for: Population- and Variant-Based Genome Analyses of Viruses from Vaccine-Derived Rabies Cases Demonstrate Product Specific Clusters and Unique Patterns
Source: Viruses. 2020 Jan 17;12(1):115. doi: 10.3390/v12010115 (PMC7020022; doi:10.3390/v12010115)
Supplement: Supplementary file 1 [file viruses-12-00115-s001.pdf]

Supplementary File

Population and variant based genome analyses of viruses from vaccine-derived rabies cases demonstrate product specific clusters and unique patterns

Sten Calvelage, Marcin Smreczak, Anna Orlowska, Conrad Martin Freuling, Thomas Müller, Christine Fehlner-Gardiner, Susan Nadin-Davis, Dirk Höper, Pawel Trebas

1.) Additional information for the preparation of overlapping amplicons of the RABV genome (2. Materials and Methods; 2.1; procedure 2)

Table S1. List of generated amplicons for the ERA BHK21-related vaccine-induced rabies cases

Sample ID	Material	Reference Method	Set of amplicons (see reference)		
C/CAN/1992N8944	Brain	[4]	A	B	C
C/CAN/1992N8991	Brain	[4]	A	B	C
C/CAN/1994N35116	Brain	[4]	A	B	C
C/CAN/1994N6762B	Brain	[4]	A	B	C
C/CAN/1994N6762M	Brain	[4]	A	B	C
C/CAN/1996N5367	Brain	[4]	A	B	C
C/CAN/1998N4916	Brain	[4]	A	B	C
C/CAN/1999N6567	Brain	[4]	A1, A2	B	C

Table S2. List of generated amplicons and the respective primer sequences used for the ERA BHK21-related vaccine-induced rabies cases

Set	Primer name	Primer sequence 5′-3′	Length	Location in ERA genome*	Sense	Amplicon Size
**A**	RVfor2	GTACGCTTAACAACAARAYCARAGAA	26	1 to 24	Pos	4374
	AFXArev	CCGGACTGACTTGTAGTGAGCATC	24	4349 - 4372	Neg	
**B**	AFXBfor	GCCCTAGAGTCCATCATGACTACCA	21	4229 - 4253	Pos	3904
	AFXBrev	GGGACATTCCAGATACCCCTCCCAA	25	8108 - 8132	Neg	
**C**	RRV-LF5a	TAACGACCAAATAGTCAACCT	24	7864-7884	Pos	4076
	RVrev2	GCGGCCGCACGCTTAACAAATAAAC	26	11915-11931	Neg	
2nd Round (as needed)						
**A1**	RVfor2	as above			Pos	2233
	#905 (#995)	CCTTAACTATGTCRTCAAGRTTCA	24	2208 - 2231	Neg	
**A2**	RRV-PF1	GAGATGGCAGAGGARACTGTAGATCT	26	1568 - 1593	Pos	2805
	AFXArev	as above			Neg	

* According to the ERA full genome sequence (NCBI accession number EF206707)

2.) Lists of samples that were considered for the population-based analysis

Table S3. Overview of vaccine batch samples used for the population-based analysis.

**Vaccine Batches**				
#	Sample ID	Vaccine Strain	Study	ENA* Study accession number (if available)
1	B1/SAD/Wistar	SAD	[1]	-
2	B/ERA/lot16 (Illumina)<br>B/ERA/lot16 (IonTorrent)	ERA BHK21<br>ERA BHK21	This study<br>[2]	PRJEB35810<br>PRJEB21530
3	B/SAD/Bern/original	$\text{Bern}_{\text{orig}}$	[1]	-
4	B/SAD/B19/744	B19	[1]	-
5	B/SAD/B19/793	B19	[1]	-
6	B/SAD/B19/806	B19	[1]	-
7	B/SAD/B19/958	B19	[2]	PRJEB21530
8	B/SAD/B19/959	B19	[2]	PRJEB21530
9	B/SAD/B19/969	B19	[2]	PRJEB21530
10	B/SAD/Bern/0213	Bern	[1]	-
11	B/SAD/Bern/4024	Bern	This study	PRJEB35810
12	B/SAD/Bern/5023	Bern	[2]	PRJEB21530
13	B/SAD/Bern/5123	Bern	[2]	PRJEB21530
14	B/SAD/Bern/7024	Bern	This study	PRJEB35810
15	B/SAD/Bern/49235	Bern	[2]	PRJEB21530

Table S4. Overview of *in vitro*-selected attenuated rabies vaccine strain batches used for the population-based analysis (not displayed in Figure 1; for more details see 2. Materials and Methods; 2.2).

***In vitro*-selected Attenuated Rabies Vaccine Strain Batches**				
#	Sample ID	Vaccine Strain	Study	ENA* Study accession number (if available)
(16)	SAD P5/88	SAD P5/88	[1]	-
(17)	SAD VA1	SAD VA1	[1]	-
(18)	SAD SAG2	SAD SAG2	[1]	-
(19)	SAD B19_{CS}	SAD B19_{CS}	[1]	-
(20)	$\text{SADB19}_{\text{P1}}$	$\text{SADB19}_{\text{P1}}$	[1]	-

Table S5. Overview of samples from vaccine-induced rabies cases used for the population-based analysis.

**Vaccine-induced rabies cases**				
#	Sample ID	Species	Study	ENA* Study accession number (if available)
1	C/CAN/1991/B	Striped Skunk	[2]	PRJEB21530
2	C/CAN/1992N8944	Red Fox	This study	PRJEB35810
3	C/CAN/1992N8991	Raccoon	This study	PRJEB35810
4	C/CAN/1994N35116	Striped Skunk	This study	PRJEB35810
5	C/CAN/1994N6762B	Cow	This study	PRJEB35810
	C/CAN/1994N6762M	Cow (MP)	This study	PRJEB35810
	C/CAN/1994/MB	Cow (MP)	[2]	PRJEB21530
6	C/CAN/1996N5367	Red Fox	This study	PRJEB35810
	C/CAN/1996/B	Red Fox	[2]	PRJEB21530
7	C/CAN/1998N4916	Raccoon	This study	PRJEB35810
8	C/CAN/1999N6567	Red Fox	This study	PRJEB35810
9	C/DEU/2002/CCS	Red Fox (CC)	[2]	PRJEB21530
10	C/AUT/2004/CCS	Red Fox (CC)	[2]	PRJEB21530
11	C/SVN/2012/CCS	Red Fox (CC)	[2]	PRJEB21530
	C/SVN/2012/CCS-2	Red Fox (CC)	[2]	PRJEB21530
12	C/SVN/2014/B	Stone Marten	[2]	PRJEB21530
13	C/LVA/2015/B	Badger	[2]	PRJEB21530
	C/LVA/2015/MBS	Badger (MP)	[2]	PRJEB21530
14	C/ROU/2015/B	Cow	[2]	PRJEB21530
15	C/POL/2017/B	Red Fox	This study	PRJEB35810

CC – Cell Culture, MP – Mouse Passage, * ENA – European Nucleotide Archive

Table S6. Overview of RABV field samples used for the population-based analysis.

**Previously investigated Field Viruses**					
#	Sample ID	No. Library	Species	Study	ENA* Study accession number (if available)
1	F/CHL/1979/CCS	1586	Human (CC)	[3]	PRJEB22369
2	F/DZA/1984/CCS	1435	Dog (CC)	[3]	PRJEB22369
3	F/SAU/1990/CCS	1681	Fox CC)	[3]	PRJEB22369
4	F/FIN/1990/CCS	1684	Fox (CC)	[3]	PRJEB22369
5	F/GER/1990/CCS	1680	Fox (CC)	[3]	PRJEB22369
6	F/ETH/1992/CCS	1682	Dog (CC)	[3]	PRJEB22369
7	F/SDN/1993/CCS	1425	Dog (CC)	[3]	PRJEB22369
8	F/KEN/2001/CCS	1589	Dog (CC)	[3]	PRJEB22369
9	F/TZA/2009/B	1440	Fox	[3]	PRJEB22369

[#]:CC – Cell Culture, * ENA – European Nucleotide Archive

Table S7. Positions of single nucleotide variants (SNVs) and their respective variant frequencies for the B/ERA/lot16 and the ERA-derived vaccine-induced rabies cases as calculated using R script for the population-based analysis [1].

Position* (Variant)	77 (A)	212 (A)	214 (C)	235 (G)	1300 (A)	1387 (A)	2043 (T)	2087 (A)	2429 (T)	2505 (T)	3085 (C)	3494 (T)	3536 (A)	3536 (G)	3587 (T)
**B/ERA/lot16 (Illumina)**	6.8	0	7.5	8.2	12.1	13.7	18.5	6.2	0	0	100	6.4	0	0	13.3
**B/ERA/lot16 (IonTorrent)**	11.6	0	6.5	6	14.1	11.8	27.7	4.9	0	0	100	5.9	0	0	11.5
**C/CAN/1991/B**	0	0	0	0	0	0	0	0	10.3	0	100	0	0	0	0
**C/CAN/1992N8944**	100	0	100	100	100	100	0	0	0	0	100	0	0	0	0
**C/CAN/1992N8991**	100	0	100	100	100	100	0	0	0	0	100	0	0	0	0
**C/CAN/1994/MB**	0	4.7	0	0	0	0	100	0	0	21.2	34.1	0	0	0	100
**C/CAN/1994N35116**	0	0	0	0	0	0	100	0	0	0	100	0	0	0	0
**C/CAN/1994N6762-B**	0	15.6	0	0	0	0	100	0	0	21	96.7	0	0	0	100
**C/CAN/1994N6762-M**	0	6.1	0	0	0	0	100	0	0	0	32.1	0	0	0	100
**C/CAN/1996/B**	100	0	100	100	100	100	0	0	0	0	100	0	56.2	43.8	0
**C/CAN/1996N5367**	100	0	100	100	100	100	0	0	0	0	100	0	43.9	56.1	0
**C/CAN/1998N4916**	0	0	0	0	0	0	100	0	0	0	100	0	0	0	100
**C/CAN/1999N6567**	0	0	0	0	0	0	0	0	0	0	100	0	0	0	0

*Nucleotide positions relative to the ERA/lot16 full genome; color schemes representing group-specific nucleotide exchanges at the consensus level (see Results 3.2; Table 4)

Table S7 (continued). Positions of single nucleotide variants (SNVs) and their respective variant frequencies for the B/ERA/lot16 and the ERA-derived vaccine-induced rabies cases as calculated using R script for the population-based analysis [1].

Position* (Variant)	**3672 (C)**	**3734 (G)**	**4720 (T)**	**5019 (C)**	**5473 (A)**	**5494 (T)**	**6917 (A)**	**7316 (C)**	**7462 (T)**	**7907 (A)**	**8063 (T)**	**8312 (T)**	**8864 (T)**	**9495 (A)**	**9534 (A)**
**B/ERA/lot16 (Illumina)**	0	8.9	17.4	8.3	38	30.5	40.2	7.3	0	0	37.1	26.6	5.4	5.7	6.2
**B/ERA/lot16 (IonTorrent)**	0	8.5	10.4	2.6	25.6	28	41	6.7	0	0	30.6	26.2	7.6	7.6	4.9
**C/CAN/1991/B**	0	0	0	0	0	100	0	0	0	0	0	100	0	0	0
**C/CAN/1992N8944**	0	0	100	0	0	0	1.2	0	0	0	0	0	0	0	0
**C/CAN/1992N8991**	0	0	100	0	0	0	0	0	0	0	0	0	0	0	0
**C/CAN/1994/MB**	5.3	0	0	0	100	0	-	0	0	0	100	0	0	0	0
**C/CAN/1994N35116**	0	100	0	0	100	0	100	0	18.4	17.4	100	0	0	0	0
**C/CAN/1994N6762-B**	0	0	0	0	100	0	100	0	0	0	100	0	0	0	0
**C/CAN/1994N6762-M**	0	0	0	0	100	0	100	0	0	0	100	0	0	0	0
**C/CAN/1996/B**	0	0	100	0	0	0	0	0	0	0	0	0	0	0	0
**C/CAN/1996N5367**	0	0	100	0	0	0	0	0	0	0	0	0	0	0	0
**C/CAN/1998N4916**	0	0	0	0	100	0	100	0	0	0	100	0	0	0	0
**C/CAN/1999N6567**	0	0	0	0	0	100	0	0	0	0	0	100	0	0	0

*Nucleotide positions relative to the ERA/lot16 full genome; color schemes representing group-specific nucleotide exchanges at the consensus level (see Results 3.2; Table 4)

Table S7 (continued). Positions of single nucleotide variants (SNVs) and their respective variant frequencies for the B/ERA/lot16 and the ERA-derived vaccine-induced rabies cases as calculated using R script for the population-based analysis [1].

Position* (Variant)	**10372 (A)**	**10491 (A)**	**10592 (T)**	**10670 (T)**	**10698 (C)**	**10840 (G)**	**10991 (A)**	**11456 (G)**	**11541 (T)**	**11583 (T)**	**11619 (G)**	**11681 (T)**	**11801 (G)**	**11825 (T)**
**B/ERA/lot16 (Illumina)**	7.2	26.2	12.5	19.5	37.7	100	16.8	5.3	35.6	0	9.3	0	6.7	0
**B/ERA/lot16 (IonTorrent)**	7.7	20	9.9	16.2	28.9	100	9.1	10.7	32.2	0	8.9	0	6.2	0
**C/CAN/1991/B**	0	0	0	0	100	100	0	0	0	0	0	0	0	5.6
**C/CAN/1992N8944**	0	0	100	100	0	33.9	100	0	0	0	0	0	0	0
**C/CAN/1992N8991**	0	0	100	100	0	100	100	0	0	0	0	0	0	0
**C/CAN/1994/MB**	0	100	0	0	0	100	0	0	100	0	0	0	0	0
**C/CAN/1994N35116**	0	100	0	0	0	100	0	0	100	0	0	6.4	0	0
**C/CAN/1994N6762-B**	0	100	0	0	0	100	0	0	100	0	0	0	0	0
**C/CAN/1994N6762-M**	0	100	0	0	0	100	0	0	100	0	0	0	0	0
**C/CAN/1996/B**	0	0	100	100	0	100	100	0	0	0	0	0	0	0
**C/CAN/1996N5367**	0	0	100	100	0	100	100	0	0	0	0	0	0	0
**C/CAN/1998N4916**	0	100	0	0	0	100	0	0	100	11.7	0	0	0	0
**C/CAN/1999N6567**	0	0	0	0	100	100	0	0	0	0	0	0	0	0

*Nucleotide positions relative to the ERA/lot16 full genome; color schemes representing group-specific nucleotide exchanges at the consensus level (see Results 3.2; Table 4

Table S8. Numbers of single nucleotide variants (SNVs) found for SAD/B19 and SAD/Bern vaccine batches

Vaccine strain	Vaccine batch	Number of SNVs	Mean
SAD/B19	B/SAD/B19/744	58	46,5
	B/SAD/B19/793	49	
	B/SAD/B19/806	45	
	B/SAD/B19/958	37	
	B/SAD/B19/959	44	
	B/SAD/B19/969	46	
SAD/Bern	B/SAD/Bern/0213	44	46,8
	B/SAD/Bern/4024	57	
	B/SAD/Bern/5023	44	
	B/SAD/Bern/5123	40	
	B/SAD/Bern/7024	58	
	B/SAD/Bern/49235	38	
Mean number of SNVs for all SAD $\text{Bern}_{\text{orig}}$-derived vaccine batches			46,7

References

1. Höper, D.; Freuling, C.M.; Müller, T.; Hanke, D.; Messling, V. von; Duchow, K.; Beer, M.; Mettenleiter, T.C. High definition viral vaccine strain identity and stability testing using full-genome population data--The next generation of vaccine quality control. *Vaccine* **2015**, *33*, 5829–5837, doi:10.1016/j.vaccine.2015.08.091.

2. Pfaff, F.; Müller, T.; Freuling, C.M.; Fehlner-Gardiner, C.; Nadin-Davis, S.; Robardet, E.; Cliquet, F.; Vuta, V.; Hostnik, P.; Mettenleiter, T.C.; et al. In-depth genome analyses of viruses from vaccine-derived rabies cases and corresponding live-attenuated oral rabies vaccines. *Vaccine* **2019**, *37*, 4758–4765, doi:10.1016/j.vaccine.2018.01.083.

3. Fischer, S.; Freuling, C.M.; Müller, T.; Pfaff, F.; Bodenhofer, U.; Höper, D.; Fischer, M.; Marston, D.A.; Fooks, A.R.; Mettenleiter, T.C.; et al. Defining objective clusters for rabies virus sequences using affinity propagation clustering. *PLoS Negl. Trop. Dis.* **2018**, *12*, e0006182, doi:10.1371/journal.pntd.0006182.

4. Nadin-Davis, S.A.; Colville, A.; Trewby, H.; Biek, R.; Real, L. Application of high-throughput sequencing to whole rabies viral genome characterisation and its use for phylogenetic re-evaluation of a raccoon strain incursion into the province of Ontario. *Virus Res.* **2017**, *232*, 123–133, doi:10.1016/j.virusres.2017.02.007.
